# Insecticidal Activity of a Component, (-)-4-Terpineol, Isolated from the Essential Oil of *Artemisia lavandulaefolia* DC. against *Plutella xylostella* (L.)

**DOI:** 10.3390/insects13121126

**Published:** 2022-12-06

**Authors:** Xing Huang, Linjie Du, Tiantian Liu, Rui Ma, Xing Liu, Haibin Yuan, Shuai Liu

**Affiliations:** Department of Plant Protection, Jilin Agricultural University, Changchun 130118, China

**Keywords:** *Plutella xylostella*, bioactive compounds, (-)-4-terpineol, insecticidal activities

## Abstract

**Simple Summary:**

*Plutella xylostella* (L.) (Lepidoptera: Plutellidae) is the most destructive pest of cruciferous crops around the world. The inappropriate use of insecticides to manage it has brought the development of resistance to several pesticide active ingredients. Keeping in view the resistance problem and long-term application, essential oils of plants may provide an environment-friendly solution to manage pests. Although the essential oil of *Artemisia lavandulaefolia* DC. was proven to exhibit promising insecticidal activities against *P. xylostella*, which components have insecticidal activities are unknown. In the present study, four bioactive components were identified from *A. lavandulaefolia* essential oil by GC/MS, and their insecticidal activities against *P. xylostella* were also evaluated. Among them, (-)-4-terpineol was proven to be mostly responsible for its insecticidal activity against *P. xylostella*. This effect was associated with decreased activity of GST, CAT, AChE and Na^+^/K^+^-ATPase. Overall, our findings indicated that (-)-4-terpineol may offer an alternative source for the control of *P. xylostella*.

**Abstract:**

*Plutella xylostella* (L.) is one of the most serious pests of cruciferous vegetables. Our previous work demonstrated that the essential oil of *Artemisia lavandulaefolia* DC. exhibits promising insecticidal activities against *P. xylostella*. This study further characterizes the key components that are responsible for the insecticidal effect. In total, 47 compounds (96.52% of the total compounds) were identified from the total oil using GC-MS, and the major compounds were eucalyptol (21.57%), D(+)-camphor (17.33%), (-)-4-terpineol (9.96%) and caryophyllene oxide (10.96%). Among them, (-)-4-terpineol showed significantly larvicidal and fumigant activities against *P. xylostella*. The LD_50_ of (-)-4-terpineol was 43.15 mg/mL at 12 h and 31.22 mg/mL at 24 h for 3rd instar larvae, and the LC_50_ for adults was 8.34 mg/mL at 12 h and 7.35 mg/mL at 24 h. In addition, the adults treated with (-)-4-terpineol showed varying degrees of inhibitory activity toward glutathione S-transferase, catalase, acetylcholinesterase and Na^+^/K^+^-ATPase at different post-treatment intervals and concentrations. The results indicate that (-)-4-terpineol has promising insecticidal activities against *P. xylostella*, and it has good inhibitory effects on the four enzymes of *P. xylostella* adults.

## 1. Introduction

The diamondback moth (DBM), *Plutella xylostella* (L.) (Lepidoptera: Plutellidae), is the most destructive pest of cruciferous crops around the world, and its control in China costs annually approximately $0.77 billion [1,2]. The extensive use of insecticides to manage it has brought the development of resistance to several pesticide active ingredients [3,4,5]. Keeping in view the resistance problem and sustainability, essential oils of plants may provide an environment-friendly solution to manage pests. Essential oils are by-products of plant metabolism that are now known to interfere with basic metabolic, physiological and behavioral functions of insects [6], thereby having promising insecticidal prospects. The components derived from essential oils were reported to be harmful for stored-grain insects. For instance, linalool, carvone, estragole and methyl eugenol obtained from coriander, caraway or basil essential oils are the most effective fumigants against *S. oryzae* [7]. The major component citronellal from aldehyde essential oil has also exhibited fumigant activity against *S. zeamais* [8] and contact insecticidal activity against *Musca domestica* [9] and *S. oryzae* [10].

The *Artemisia* genus belongs to the important family Compositae (Asteraceae), one of the most numerous plant groupings, which comprises about 1000 genera and over 20,000 species. Within this family, *Artemisia* is included in the tribe Anthemideae and comprises over 500 species [11], among which approximately 185 species are found in China [12]. Many species have been used as traditional Chinese medicinal herbs for treating diseases such as malaria, cancer, hepatitis, inflammation, and autoimmune diseases [13,14,15]. *Artemisia lavandulaefolia* DC. (Asteraceae) is a perennial herb with a strong aroma. Previous investigation showed that the essential oil from *A. lavandulaefolia* has significant biological activity against *P. xylostella* adults, which provided 80% to 100% repellency at a 1% *v/v* concentration [16]. Furthermore, the fumigation activity of this essential oil against *Sitophilus zeamais* (Motsch) (Coleoptera: Curculionidae) was assessed, at a dose of 0.10 uL, and the corrected mortality caused was 73.46% [17], whereas its LC_50_ value was 31.81 mg/L air when used against adults of *Lasioderma serricorne* (Fleming) (Coleoptera: Anobiidae) [18].

The components of essential oil from *A. lavandulaefolia* mainly consist of monoterpenes, sesquiterpene hydrocarbons, sesquiterpenes and flavonoids [19,20]. For instance, caryophyllene, cineole, eucalyptol, borneol, *α*-terpineol and *β*-farnesene have been identified [17,21,22]. The chemical constituents of *A. lavandulaefolia* essential oil differ due to seasonal geographic factors and the extraction method [23]. However, reports on the main constituents from *A. lavandulaefolia* essential oil that have insecticidal effects are scarce. In the present study, four major components, eucalyptol, D(+)-camphor, (-)-4-terpineol and caryophyllene oxide were identified from *A. lavandulaefolia* essential oil by GC/MS, and their insecticidal activities against *P. xylostella* were also evaluated. Among them, (-)-4-terpineol is widely distributed in *Melaleuca alternifolia* and *Origanum majorana* [24]. Recently, research on (-)-4-terpineol has mainly focused on its medicinal properties [25,26,27,28]. It also demonstrated insecticidal activity against stored grain and sanitary pests [29], such as the red flour beetle (*T. castaneum*) [30,31], *S. oryzae* [29], human head louse (*Pediculus humanus capitis*) [32], house dustmites (*Dermatophagoides farinae* and *Tyrophagus putrescentiae*) [33] and termites (*Reticulitermes speratus*) [34]. Whether (-)-4-terpineol has insecticidal activity against *P. xylostella* is unknown.

The insecticidal activity of the essential oils has been associated with their impact in the changes in enzyme activity in vivo. Enzymes play an important role in the detoxification and metabolism of exogenous toxic substances in insects. Glutathione-S-transferase (GST) is the detoxification enzyme that is generally involved in the metabolism of deleterious secondary plant metabolites in botanical insecticides. This enzyme also is enhanced by involvement in the detoxification of xenobiotic compounds. For example, the enhancement level of GST was demonstrated after *Tuta absoluta* (Meyrick) (Lepidoptera: Gelechiidae) treatment with Ajwain essential oil and its constituents [35]. However, due to differences in the detoxification ability of insects, the essential oil extracted from Chinese chive inhibited the activity of GST in treated *P. xylostella* larvae at the 50% lethal concentration [36].

In addition to the detoxifying enzyme, essential oils of plants inflict several physiological processes, including antioxidant enzymes, such as catalase (CAT), which protects organisms against oxidative stresses [37]. Also, AChE is an important enzyme in biological nerve conduction ions, the activity of AChE is inhibited to a certain extent and may directly cause insect paralysis and death [38]. Na^+^/K^+^-ATPase is widely present on the cell membrane of various organisms, maintaining the potential balance on both sides of the cell membrane, thereby ensuring the smooth and continuous process of nerve impulses. The essential oils from *Santolina chamaecyparissus* L., *Achillea millefolium* L., *Tanacetum vulgare* L., *Tagetes patula* L. and *A. absinthium* L. were reported to act as the AChE inhibitor and blocked the Na^+^/K^+^-ATPase of *Myzus persicae* (Sulzer) (Hemiptera: Aphididae) [39]. From the phenomenon of insect poisoning, the mode of action of essential oil compounds elicits characteristic neurotoxic symptoms including agitation, hyperactivity, paralysis and knockdown. Thus, the objective of our work was to assess the insecticidal activity of eucalyptol, D(+)-camphor, (-)-4-terpineol and caryophyllene oxide against larvae and adults of *P. xylostella* and also to determine the impact of the active essential oil compound (-)-4-terpineol on the GST, CAT, AChE and Na^+^/K^+^-ATPase enzyme activities of the diamondback moth.

## 2. Materials and Methods

### 2.1. Insects

*Plutella xylostella* was obtained from the experimental field of Jilin Agricultural University, Changchun, China, and then was maintained in a screen cage (43 × 43 × 43 cm). Larvae were reared on *Brassica oleracea* var. *capitata* that had never been exposed to pesticides. Enclosed adults were fed on 10% honey water as a nutritional supplement. The setup was arranged in a plant growth chamber at 25 ± 1 °C, 65 ± 5% RH, and a photoperiod of 16:8 (light:dark cycle).

### 2.2. Plant Materials and Essential Oil Extraction

The aerial parts of *A. lavandulaefolia* were previously identified and confirmed by Dr. Tong-Bao Qu (Voucher specimen: JLH 2158), College of Horticulture, Jilin Agricultural University. The aboveground parts, including stems, leaves and flowers, were collected at the end of August 2019 in Changchun (43.8170° N, 125.3235° E). The harvested parts were cut into pieces and then dried in the shade for 10 days. Each 1.5 kg of dried plant material was soaked with water for 12 h. Then, the samples were subjected to hydrodistillation for 2 h using plant essential oil extraction equipment (Shanghai Yuyan Machinery Equipment Co., Ltd. Shanghai, China). The obtained essential oil was stored in amber sealed glass bottles for refrigeration at 4 °C until use.

### 2.3. Authentic Compounds

(-)-4-terpineol (liquid, 98% purity) was purchased from Tokyo Chemical Industry (Tokyo, Japan), eucalyptol (liquid, 99% purity) and D(+)-camphor (solid, 96% purity) were purchased from Aladdin (Shanghai, China) and caryophyllene oxide (solid, 95% purity) was purchased at Acros Organics (Geel, Belgium). The reagents used in the study were obtained from Beijing Chemical Industry, Beijing, China. Cypermethrin (solid, 99.0% purity) and dichlorvos (liquid, 95% purity) were purchased from Sigma-Aldrich (St. Louis, MO, USA).

### 2.4. Identification of Compounds

The *A. lavandulaefolia* essential oil composition was analyzed by gas chromatography using a GC system (Agilent 6890N, Agilent Technologies Incorporated, Santa Clara, CA, USA). An HP-1 capillary column (30 m × 0.25 mm × 0.25 μm film thickness) was used with injection in split-ratio mode (40:1). The oven temperature started at 60 °C for 3 min, and then it was increased to 100 °C (10 °C/min). Afterward, it was increased to 180 °C (5 °C/min) and subsequently increased to 280 °C (15 °C/min), and remained for 5 min. The carrier gas was helium at a flow rate of 1.0 mL/min, and the injection volume used in each analysis was 0.5 μL.

The mass spectrometer (Agilent 5975N, Agilent Technologies Incorporated, Santa Clara, CA, USA) used an electron ionization source with 70 eV ionization energy. The ion source temperature was 230 °C, with a scanning range between 20 and 800 *m/z*. The temperature of the quadrupole was 150 °C, and the mass spectrum acquisition delay time was 4 min. The constituents were identified based on their retention index and confirmed by the use of mass spectral libraries (National Institute of Standards and Technology, NIST databases). In addition, the major oil compounds were identified by co-injection with standards. The relative concentration of each component in the oil was quantified by a peak area normalization method integrated into the analysis.

### 2.5. Toxicity Bioassays of Bioactive Compounds

To assess the insecticidal activity of the identified four main bioactive compounds, insect mortality was determined through contact and fumigant bioassays.

#### 2.5.1. Contact Bioassays

Each main compound and the essential oil were diluted in acetone to yield a concentration of 100 mg/mL, and 1 μL of each preparation was dropped on the dorsal thoracic region of larvae. Treatment with acetone was used as a blank control. Ten larvae (3rd instar) were used for treatment in a glass petri dish (90 × 20 mm in diameter), and five replicates were made. All topically treated individuals were maintained in incubators (25 ± 1 °C, 70 ± 5%, RH, 16 L: 8 D), and larval mortality was recorded after 12 and 24 h. The larvae were judged to be dead if there was no reaction to touching the body with a brush. Cypermethrin served as a positive control.

#### 2.5.2. Fumigant Bioassays

Three-day-old adult insects were assessed for fumigant toxicity. The chemicals and essential oil were prepared at a dose of 15 mg/mL using acetone. Application of acetone was used as a blank control. Dichlorvos was used for positive control. 10 μL of each sample was placed on filter paper (10 × 1.5 cm), which was placed in a 60-mL vial with ten adults, and the solvent evaporated after 15 s. Then, the vial was sealed immediately. All experiments were performed in incubators (25 ± 1 °C, 70 ± 5%, RH, 16 L: 8 D) for observation at 12 and 24 h. Five replicates were conducted. The adults were considered dead if they were unresponsive when the bottles were shaken.

### 2.6. Median Lethal Dose (LD_50_) and Concentration (LC_50_)

To evaluate the insecticidal activity of (-)-4-terpineol, serial dilutions were prepared at different concentrations (10, 20, 30, 50, 70 mg/mL) using acetone for the contact toxicity bioassay. For the fumigant toxicity bioassay, concentrations of 5, 7, 10, 15 and 30 mg/mL were prepared separately for evaluation. Mortality was determined after 24 h and 48 h.

### 2.7. Median Knockdown Time (KT_50_) of (-)-4-Terpineol

10 μL of (-)-4-terpineol dissolved in acetone was dropped onto filter paper at the LC_30_, LC_50_ and LC_80_ concentrations. After the solvent evaporated (15 s), they were placed in a 60-mL glass vial that contained ten adults. As controls, ten adults were treated with acetone alone in each case. Insects lying on the bottom that were unable to walk or fly were considered knocked down, and the number of knocked-down insects was registered at every 15 s for 15 min. In general, each replicate was interrupted when >90% of the insects were affected. Five independent replicates were carried out for each concentration.

### 2.8. Enzyme Assays

#### 2.8.1. Enzyme Preparation Method

Among four components, (-)-4-terpineol expressed significant insecticidal activity against *P. xylostella* adults. Hence, the levels of enzyme activities in adult exposure to (-)-4-terpineol were investigated. In the fumigant bioassays, adults obtained at 0.5 h, 2 h, 4 h, 8 h and 12 h post treatment with (-)-4-terpineol at the LC_30_, LC_50_, and LC_80_ levels were subjected to enzyme preparation to determine the detoxification and neuron enzyme activities. Ten adults were placed in a glass homogenate tube and homogenized in 2 mL ice-cold normal saline. The homogenate was centrifuged at 4000 rpm (6887 g) for 10 min at 4 °C (centrifuge 5430R, Eppendorf, Hamburg, Germany), and the supernatant was used as an enzyme source for enzyme assays.

#### 2.8.2. Protein Assay

The protein concentration was measured using the method of Bradford (1976) by adding 5 μL of adult homogenate to 15 μL phosphate buffer (0.1 M, pH 7.2), and then 200 μL of Bio-Rad reagent was added in triplicate. After incubation of the mixture for 10 min at room temperature, the absorbance was measured of 200 μL in a 96-well microplate reader (SpectraMax i3x Multi-Mode Microplate Reader, Molecular Devices, Sunnyvale, CA, USA) at 590 nm. A bovine serum albumin standard curve obtained with the same method and reagents were used to convert the absorbance to protein concentration.

#### 2.8.3. GST Activity Assay

The test solution was prepared from the supernatant (90 μL), pH 7.2 potassium phosphate buffer (0.1 M, 810 μL), glutathione solution (20 mM GSH reduced form, 45 μL) and 1-chloro-2, 4′-dinitrobenzene (CDNB, 20 mM, 45 μL). The GST activity of the mixture was determined using an ultraviolet-visible spectrophotometer (UV759CRT, Shanghai Youke Instrument Co., Ltd., Shanghai, China) at 340 nm in triplicate for 3 min. A total of 90 μL of distilled water was used as a blank control.

#### 2.8.4. CAT Activity Assay

Preparation of H_2_O_2_ solution: Firstly, 0.5 mL of 30% H_2_O_2_ was taken to 50 mL of distilled water, phosphate buffer (0.05 M, pH 7.4) was added to the front-matched solution to measure the absorbance of this solution between 0.5 and 0.55 at 240 nm. Subsequently, the reaction mixtures contained 3 mL of the prepared solution and 20 μL of enzyme solution. The CAT activity of the mixture was recorded for 3 min with a spectrophotometer, and the experiment was performed in triplicate.

#### 2.8.5. AChE Activity Assay

The reaction solution was prepared from supernatant (90 μL), pH 7.2 potassium phosphate buffer (0.1 M, 720 μL), acetylthiocholine iodide (20 mM, 45 μL) and 5, 5′-dithiobis-(2-nitrobenzoic acid) (DTNB, 20 mM, 45 μL). The AchE activity in mixture was calculated using a spectrophotometer for 3 min at 412 nm. Distilled water was used as a blank control. The experiment was also conducted in triplicate.

#### 2.8.6. Na^+^/K^+^-ATPase

Enzyme assay kits (Nanjing Jiancheng Bioengineering Institute, Nanjing, China) were used to measure the Na^+^/K^+^-ATPase activities of the enzymes. Briefly, the reaction mixtures contained prepared enzyme solution (0.1 mL), distilled water (0.12 mL) and working fluid (0.56 mL). Approximately 0.16 mL distilled water and 0.42 mL working fluid were mixed together to serve as blank controls. After incubating the mixtures at 37 °C for 10 min, the mixtures were centrifuged at 3500 rpm for 10 min to obtain a supernatant. The test solution contained 0.3 mL of prepared supernatant, 1.0 mL chromogenic agent and 1 mL working fluid. A total of 200 μL of the solution was added to a 96-well plate in each test, and the absorbance of each well was measured at 636 nm. Each sample, control, standard, and blank measurement was tested in triplicate, and each test was performed in triplicate.

### 2.9. Statistical Analysis

The insect mortality rates were corrected for mortality against the control using Abbott’s formula [40], and the LD_50_, LC_50_ and KT_50_ values of the chemicals were analyzed using probit analysis [41]. All data were analyzed using SPSS Statistics 17.0 (SPSS, Chicago, IL, USA). The differences between the groups were statistically compared by one-way ANOVA and Tukey’s HSD test at 5% significance. Analysis of 50% mortality (LC_50_, LD_50,_ and KT_50_) resulting from treatment with (-)-4-terpineol was calculated by log-probit analysis containing 95% confidence limits. These data were plotted by GraphPad Prism 5.0 (GraphPad Software, La Jolla, CA, USA).

## 3. Results

### 3.1. Composition of the Essential Oil

**Figure 1:**
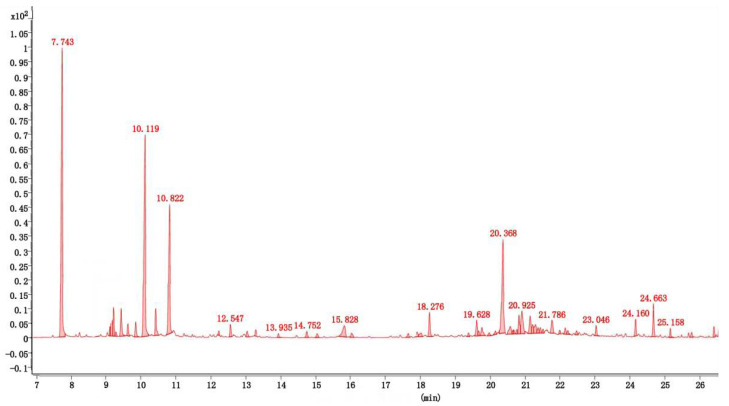
Total iron current chromatogram of the essential oil from *A. lavandulaefolia*.

The composition of the extracted essential oil is shown in Table 1 and Figure 1. Forty-seven compounds were identified, which accounted for 96.52% of the total essential oil. Among them, four major compounds accounted for 59.82% of the total oil by GC–MS analysis, including eucalyptol (21.57%), D(+)-camphor (17.33%), (-)-4-terpineol (9.96%), and caryophyllene oxide (10.96%). Therefore, the biological activities of these four compounds were tested in the next experiment.

### 3.2. Insecticidal Assays

**Figure 2:**
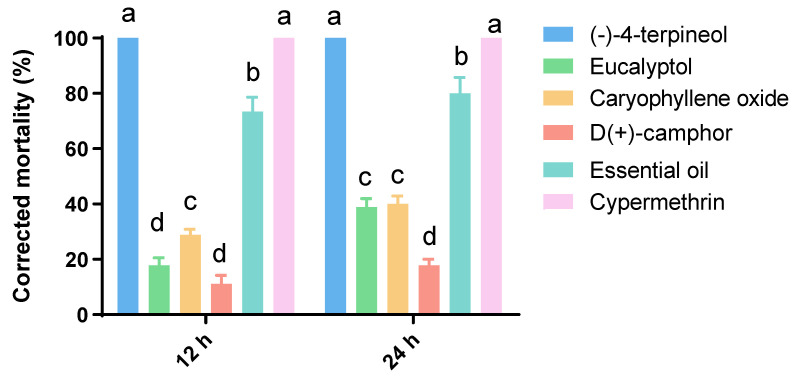
Toxicity of major compounds of *A. lavandulaefolia* essential oil against *P. xylostella* larvae in comparison to whole essential oil and positive control by contact bioassays after 12 h and 24 h of topical application. The values are expressed as the means + standard error of each experiment. Bars followed by the same letter in each time interval are not significantly different using Tukey’s HSD test at *p* < 0.05.

In the assessment of contact activity against the third instar larvae of *P. xylostella*, the corrected mortality for compounds, essential oil and positive control were determined after 12 h and 24 h. No mortality was observed in the control group. As shown in Figure 2, (-)-4-terpineol showed the best insecticidal activity among the four compounds, which had the same effect as the positive control (cypermethrin). The corrected mortality reached 100% at 12 h post treatment. Furthermore, the insecticidal effect of (-)-4-terpineol was significantly (F = 32.00, *df* = 5, 16, *p* < 0.01) better than that of the essential oil.

**Figure 3:**
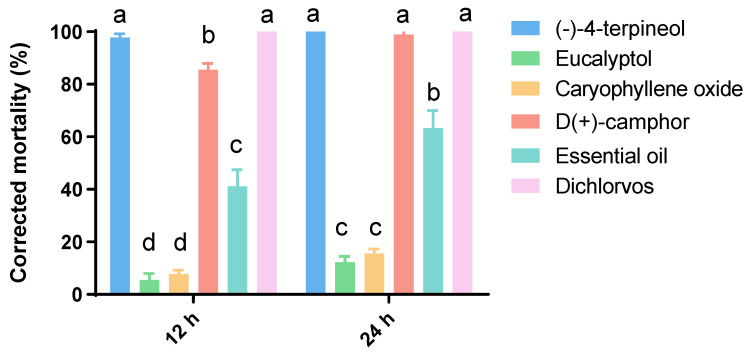
Toxicity of major compounds of *A. lavandulaefolia* essential oil against *P. xylostella* larvae in comparison to the essential oil and the positive control, 12 h and 24 h after fumigation. The values are expressed as the means + standard error of each experiment. Bars followed by the same letter in each time interval are not significantly different using Tukey’s HSD test at *p* < 0.05.

The insecticidal activity against *P. xylostella* adults of the four compounds and essential oil were also tested by fumigant toxicity bioassays (Figure 3). Similar to the results of contact activity, (-)-4-terpineol showed the best insecticidal activity against *P. xylostella* adults, which resulted in 100% insect mortality at 24 h. Additionally, the insecticidal effect of (-)-4-terpineol was significantly (F = 35.56, *df* = 5, 16, *p* < 0.01) better than that of the essential oil. The control had no insect mortality, and the positive control (dichlorvos) also exhibited 100% mortality. Meanwhile, the D(+)-camphor recorded 100% insect mortality at 24 h, but it was not significantly different from that of (-)-4-terpineol at 12 h exposure. During the experiment, it was found that a large number of test adults treated with essential oil were knocked down in the early stage, but they mostly woke up after 8 h of treatment. Thus, the essential oil showed 41.11% mortality at 12 h.

### 3.3. Toxicity Bioassays

**Figure 4:**
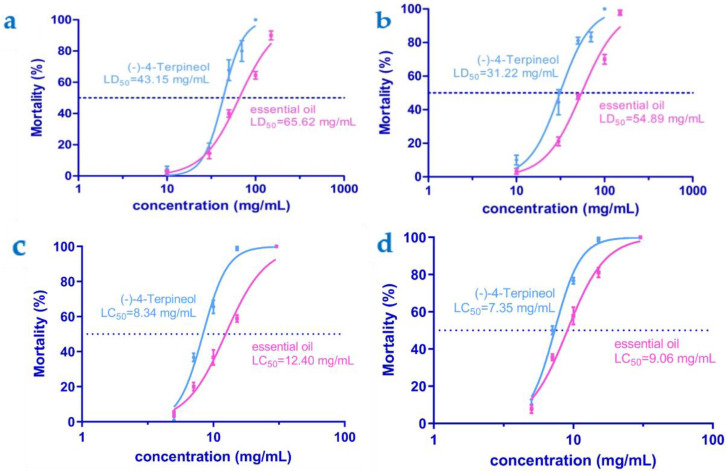
Contact toxicity (**a**,**b**) of (-)-4-terpineol and the essential oil of *A. lavandulaefolia* against *P. xylostella* larvae and their fumigant toxicity (**c**,**d**) against *P. xylostella* adults, at 12 and 24 h post treatment, respectively. Vertical bars represent standard error of the mean. The curves have been drawn from the insect mortality at five concentrations using GraphPad Prism 5.0. Lethal dosage (LD) and lethal concentration (LC) values were estimated based on a concentration–mortality bioassay using probit analysis. LD_50_: Lethal dosage that kills 50% of the exposed larvae, expressed in mg/mL. LC_50_: Lethal concentration that kills 50% of the exposed adults, expressed in mg/mL.

Bioassays of individual compounds revealed that (-)-4-terpineol was significantly more active than the whole essential oil. As for the contact toxicity bioassays, (-)-4-terpineol also had promising considerable larvicidal activity against the larvae of *P. xylostella* with LD_50_ = 43.15 (36.93 to 50.41) mg/mL at 12 h and 31.22 (24.36–40.00) mg/mL at 24 h post treatment, respectively (Figure 4a,b). The results showed that (-)-4-terpineol was more active against *P. xylostella* adults in the fumigant bioassays than in the contact toxicity (Figure 4c,d), with LC_50_ values of 8.34 (7.40–9.40) mg/mL at 12 h and 7.35 (6.69 to 8.09) mg/mL at 24 h post treatment.

### 3.4. Median Knockdown Time (KT_50_) of (-)-4-Terpineol

After being fumigated by (-)-4-terpineol, the test insects were knocked down firstly and lost their ability to fly normally. The different concentrations showed different levels of bioactivities against the adults (Table 2). After treatment with the LC_30_, LC_50_ and LC_80_ concentration, the KT_50_ values were 9.50, 6.07 and 5.09 min, respectively. Adults in the control group flew normally in the bottle with no knockdown phenomenon. Generally, KT_50_ values decreased with increasing (-)-4-terpineol concentration and thus the susceptibility of adult *P. xylostella* was directly associated with (-)-4-terpineol concentration as well as the time of exposure.

### 3.5. Enzyme Activities Assays

**Figure 5:**
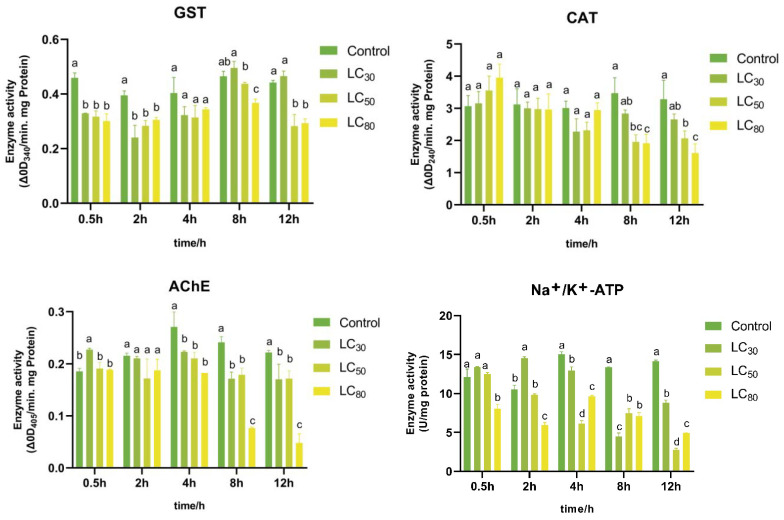
Effect of (-)-4-terpineol on enzyme activities of *P. xylostella* adults after fumigation. In each enzyme activity chart, bars followed by the same letter in each time interval are not significantly different using Tukey’s HSD test at *p* < 0.05. Units used: GST (Glutathione S-transferase): CDNB conjugated product/mg protein/min, CAT (Catalase): Remaining amount of H_2_O_2_/mg protein/min, AChE (Acetylcholinesterase): 1.3.5-trinitrobenzene (TNB) product/mg protein/min, Na^+^/K^+^-ATPase: 1 μmol inorganic phosphorus product/mg protein.

The enzyme activities in *P. xylostella* adults treated with compound (-)-4-terpineol are presented in Figure 5. The GST enzyme activities of the *P. xylostella* were significantly decreased when they were treated with (-)-4-terpineol for 2 h, and then they increased after 8 h of exposure. Finally, we found that GST activity in specimens treated with LC_50_ and LC_80_ was decreased in comparison to the control after 12 h exposure, and the GST levels were 0.64-fold and 0.66-fold, respectively, relative to the control.

CAT activity did not show any significant inhibition for the first 4 h. After 8 h of exposure, the CAT activities decreased significantly in the different treatments, and the bioactive compound had a greater effect on the enzyme activity as the concentration increased. Moreover, compared with those in the control group after 12 h of application, the CAT expression levels were 0.81-fold, 0.63-fold, and 0.49-fold at concentrations of LC_30_, LC_50_ and LC_80_, respectively.

In the first 2 h, compared with the control, the AChE activities showed no effect when treated with (-)-4-terpineol at LC_50_ and LC_80_, except the concentration of LC_30_ at 0.5 h, which was increased. High-concentration treatment (LC_80_) had the greatest inhibitory effect on the enzyme viability by 0.22-fold after exposure for 12 h.

The compound (-)-4-terpineol at different concentrations could affect the Na^+^/K^+^-ATPase activity of *P. xylostella* adults via the induction or inhibition of enzyme activities. Those treated with LC_30_ showed a significant increase in comparison to the control after 2 h. After 4 h of exposure, all concentration treatments significantly decreased the Na^+^/K^+^-ATPase activity. Among the four enzyme activities, (-)-4-terpineol has a high degree of inhibition of Na^+^/K^+^-ATPase activity. Likewise, compared with those in the control group after 12 h, the expression levels of Na^+^/K^+^-ATPase at LC_30_, LC_50_ and LC_80_ were inhibited rapidly by 0.62-fold, 0.20-fold, and 0.35-fold, respectively.

## 4. Discussion

Essential oils are natural complex secondary metabolites [42]. Most essential oils comprise monoterpenes (C_10_), sesquiterpenes (C_15_), and higher terpenes. Previous study showed that essential oils and their constituents have insecticidal properties against diverse pests [43,44,45,46]. For instance, carvone, menthol, cineole [47], thymol, 1,8-cineole, terpineol, carvacrol, linalool [48], and citronellal [10] have been evaluated as fumigants against *Tribolium castaneum* Herbst (Coleoptera: Tenebrionidae), and cineole also had contact toxicity against this pest [47]. The essential oil from *A. lavandulaefolia* was isolated in our study. The main components were eucalyptol, D(+)-camphor, (-)-4-terpineol and caryophyllene oxide. To evaluate whether there was insecticidal toxicity of four compounds identified from the essential oil, an experiment using pure samples was carried out. The results revealed that (-)-4-terpineol showed a significantly stronger insecticidal activity against *P. xylostella* via contact and fumigant toxicity than the other compounds. Also, the D(+)-camphor recorded 100% insect mortality at 24 h by fumigant bioassay. Previous study demonstrated that D(+)-camphor showed strong fumigant toxicity against *Spodoptera litura* (Fabricius) (Lepidoptera: Noctuidae) [49]. Furthermore, eucalyptol and caryophyllene oxide were less active to *P. xylostella*; however, it is possible that they may be effective at higher doses. The fumigation effect of the two main constituents from *Chenopodium ambrosioides* L. essential oil, a-terpinene and p-cymene, against *P. xylostella* larvae was determined with LC_50_ values of 284.6225 and 221.6663 mg/mL, respectively [50]. Our study demonstrated that (-)-4-terpineol showed stronger toxicity in the fumigant bioassay, and the corrected mortality reached 100% at a dose of 15 mg/mL; the LC_50_ was 8.34 mg/mL at 12 h of exposure and 7.35 mg/mL at 24 h of exposure. After treatment with the LC_50_ concentration, the adults were hyperactive and the KT_50_ value was only 6.07 min. To the best of our knowledge, not much work on the insecticidal activities of the (-)-4-terpineol has been performed. Furthermore, in our study, the bioassays of the major component (-)-4-terpineol showed that it was significantly more active than the crude essential oil. Thus, it can be inferred that (-)-4-terpineol may act as the major compound with good insecticidal potential among the four compounds. Consistently, the *trans*-*p*-Mentha-1(7), 8-dien-2-ol showed stronger insecticidal toxicity than essential oil and other compounds by contact and fumigant toxicity [51]. Similar observations have been reported by Muturi et al. [52], who found that the single constituent patchouli alcohol, identified from honeysuckle essential oil, was significantly more toxic against *Aedes aegypti* (L.) (Diptera: Culicidae) than the whole essential oil and its other constituents. However, a variety of documents suggest that complex mixtures would be more efficient and synergistic effects have been reported [53]. It is demonstrated that thymol and α-terpineol synergized the impacts of both linalool and 1,8-cineole, but linalool with 1,8-cineole exhibited only an additive effect against *Chilo partellus* (Swinhoe) (Lepidoptera: Pyralidae) [54]. Consequently, future research needs to focus on developing unique mixtures that can serve as acute toxicant. Enzymes in insects play important roles in biochemical reactions and physiological effects in toxicological studies [55]. Therefore, the effects on GST, CAT, AChE and Na^+^/K^+^-ATPase were studied based on the highest toxicity of (-)-4-terpineol in this study. Glutathione-S-transferases are well-known detoxification enzymes that metabolize potentially toxic substances to reduce toxicity [56]. In this study, the GST enzyme activity showed a decreasing trend within 2 h exposure, which showed that the detoxification enzyme in *P. xylostella* started to work after exposure to (-)-4-terpineol, and then it could not be detoxified in the insect body and appeared to be inhibited. Similar results were reported by Liao et al. [57], who found that the activities of GST in *Helicoverpa armigera* (Hübner) (Lepidoptera: Noctuidae) were notably inhibited by *Melaleuca alternifolia* essential oil compared with the control. This may be due to the low detoxification ability of the pests against these exogenous compounds at the beginning. Afterwards, (-)-4-terpineol induced the detoxification mechanism of *P. xylostella* after 4 h of treatment, which led to the activity of GST increasing for a short time and then decreasing; the reduced enzyme activity at later stages might be due to the long-term effects of the compound. In another study, the GST activity decreased in *P. xylostella* larvae that were treated with Chinese chive essential oil at LC_50_ concentration after 24 h treatment relative to the controls [36]. Furthermore, catalase (CAT) is an important antioxidant enzyme in insects [37]. In our study, there was no effect on CAT activity in the early stage. However, it was inhibited significantly in different concentrations after 8 h of exposure, and the component used to treat insects notably inhibited the enzyme activity as the concentration increased, which is in alignment with the results of Rajkumar et al. [58] and Kiran and Prakash [59]. A significant decrease in CAT activity was observed for the essential oils of *Mentha piperita* and *Gaultheria procumbens* and their major compounds (menthone, menthol and methyl salicylate) when applied to three storage pests [60]. The fumigant toxicity of (-)-4-terpineol may be related to the inhibition of AChE due to the hyperactivity and knockdown effects observed in the experiments. Neural excitation and knockdown effects are the result of impairment in neuromuscular behavior [57]. Monoterpenoids in essential oils inhibited AChE activity, as reported by Miyazawa and Yamafuji [61]. Our current study revealed that the bioactive component (-)-4-terpineol increased the AChE activity of *P. xylostella* at sublethal concentrations at the early stage, whereas other concentrations made no difference in comparison to the control. Then, they were inhibited later. Higher AChE inhibitory effects were observed in the high-concentration treatment (LC_80_), which inhibited enzyme viability by 0.22-fold relative to the control after exposure for 12 h. This result implied that the inhibition of AChE may lead to the accumulation of acetylcholine at neuromuscular junctions, which may in turn induce neuronal excitation, hyperactivity, paralysis, and ultimately result in insect death [62]. Furthermore, we investigated the possible involvement of another biochemical target (Na^+^/K^+^-ATPase), which is an important enzyme in the insect nervous system. Na^+^/K^+^-ATPase is the ion pump of Na^+^ and K^+^ in the epicyte of insects, which plays a crucial role in maintaining the ionic balance and nerve impulse of the insect body [63]. We observed that the insects were flying with excitement and twitching their heels and beaks during treatment. Moreover, our work ascertained that (-)-4-terpineol at different concentrations could significantly inhibit the Na^+^/K^+^-ATPase activity of *P. xylostella*. In particular, the expression level of Na^+^/K^+^-ATPase at LC_50_ was inhibited rapidly by 0.20-fold compared with those in the control group after 12 h exposure. The implications of these results are that the enzyme inhibitory effect of (-)-4-terpineol may result in nerve aberration, metabolic disturbances, knockdown toxicity and insect mortality. Another study documented that *Piper sarmentosum* essential oil and myristicin showed a strong inhibiting effect on the activity of Na^+^/K^+^-ATPase in *Brontispa longissima* (Gestro) (Coleoptera: Hispidae) larvae [63]. All nine terpene derivatives identified from *Citrus sinensis* essential oil caused a pronounced reduction in this enzyme in *Callosobrunchus maculatus* (Fabricius) (Coleoptera: Bruchidae) adults, and the reduction in enzyme activity was in a dose-dependent manner [64]. Although these bioactive compounds have different effects on different enzymes, their effects on the enzyme activities are still very complicated because these components may accumulate together and work synergistically in vivo. Overall, enzymes in pests exposed to (-)-4-terpineol were inhibited. Thus, these findings revealed that the biochemical reaction/physiological disturbances of pests may be involved in the toxic effects of (-)-4-terpineol on insects.

## 5. Conclusions

In conclusion, the investigation of chemical components from natural products is vital to the development of new insecticides. Based on the results presented in this work, a bioactive ingredient of *A. lavandulaefolia* essential oil, (-)-4-terpineol, showed promising insecticidal activity against *P. xylostella*. This effect was associated with decreased activity of GST, CAT, AChE and Na^+^/K^+^-ATPase. In the future, the detailed mechanisms of action of (-)-4-terpineol were necessary to be explored. Also, studies on the insecticidal efficiency of (-)-4-terpineol under the field conditions are needed.

## Figures and Tables

**Table 1:** Chemical constituents and relative contents of the essential oil from *A. lavandulaefolia*.

Retention Time/min	RI ^2^	Name of Constituent	Relative Content/%	Identifaicaion Methods	CAS ^4^
7.743	1022	Eucalyptol	21.57	MS ^1^, RI	470-82-6
8.7181	1072	Artemisia alcohol	3.90	MS, RI	27644-04-8
9.1215	1083	Nonanal	0.42	MS, RI	124-19-6
9.1685	1119	Filifolone	3.20	MS, RI	4613-37-0
9.2205	1089	Thujone	1.59	MS, RI	546-80-5
9.4407	1149	Chrysanthenol	1.95	MS, RI	18383-58-9
9.6387	1103	2-pinen-7-one	0.80	MS, RI	473-06-3
9.8565	1163	Isocyclocitral	1.05	MS, RI	1335-66-6
10.1188	1121	D(+)-camphor	17.33	MS, RI	464-49-3
10.4331	1147	*cis*-chrysanthenol	1.77	MS, RI	55722-60-6
10.8217	1160	(-)-4-terpineol	9.96	MS, RI, Co ^3^	20126-76-5
10.943	1171	(-)-myrtenal	0.27	MS, RI	564-94-3
12.2175	1230	3-Isopropylbenzaldehyde	0.25	MS, RI	34246-57-6
12.9526	1190	(+)-isopiperitenone	0.29	MS, RI	16750-82-6
13.0268	1252	perillyl aldehyde	0.47	MS, RI	2111-75-3
13.2718	1273	(-)-Bornyl acetate	0.43	MS, RI	5655-61-8
13.9351	1279	(E,Z)-2,4-decadienal	0.26	MS, RI	25152-83-4
14.7519	1333	Terpinyl acetate	0.45	MS, RI	80-26-2
15.8285	1120	3-caren-5-one	1.82	MS, RI	81800-50-2
16.0314	1372	Methyl eugenol	0.42	MS, RI	93-15-2
17.6526	1457	4-(2,2,6-trimethyl-bicyclo [4.1.0]hept-1-yl)-butan-2-one	0.35	MS, RI	77143-20-5
17.9199	1586	Zizanol	0.35	MS, RI	28102-79-6
18.2764	1325	Agropyrene	1.69	MS, RI	520-74-1
19.3952	1521	Cabreuva oxide	0.25	MS, RI	107602-52-8
19.6278	1577	*β*-Caryophyllene	1.18	MS, RI	87-44-5
19.7813	1578	*β*-caryophyllene alcohol	0.83	MS, RI	58404-89-0
19.9868	1563	Palustrol	0.29	MS, RI	5986-49-2
20.1625	1419	Caryophyllene	0.28	MS, RI	69842-07-5
20.3679	1574	Caryophyllene oxide	10.96	MS, RI	1139-30-6
20.5858	1584	Salvial-4(14)-en-1-one	0.32	MS, RI	73809-82-2
20.8358	1534	Copaborneol	0.57	MS, RI	21966-93-8
21.1576	1613	Junenol	1.37	MS, RI	472-07-1
21.2269	1688	8-cedren-13-ol	0.67	MS, RI	18319-35-2
21.3135	1540	Selina-3,7(11)-diene	0.93	MS, RI	6813-21-4
21.3803	1631	Longifolenaldehyde	0.40	MS, RI	19890-84-7
21.4521	1376	Copaene	0.40	MS, RI	3856-25-5
21.5313	1604	Widdrenal	0.26	MS, RI	470-41-7
21.6427	1873	(2-Butyrylphenoxy) acetic acid	0.27	MS, RI	65627-57-8
21.7863	1621	Desmethoxyencecalin	1.31	MS, RI	19013-07-1
22.1601	1324	10,10-Dimethyl-4-acetyl-tricyclo [5.2.1.0(1,5)]decane	0.45	MS, RI	176171-97-4
22.4843	1668	Bisabolol	0.33	MS, RI	515-69-5
24.1602	1710	4,5-Dichloro-2-octyl-isothiazolone	1.04	MS, RI	64373-81-5
24.6627	1810	Methyl isocostate	1.92	MS, RI	132342-55-3
25.1578	1842	Fitone	0.51	MS, RI	502-69-2
25.7718	1724	Euparine	0.36	MS, RI	532-48-9
26.4055	2513	Cinerin II	0.60	MS, RI	121-20-0
26.5491	1236	β-homocyclocitral	0.43	MS, RI	472-66-2

^1^ MS, mass spectrum. ^2^ RI, retention index, as determined on an HP-1 MS capillary column using the homologous series of *n*-hydrocarbons. ^3^ Co, co-injection with authentic compound. ^4^ CAS, Chemical Abstract Service.

**Table 2:** 50% knockdown times caused by (-)-4-terpineol dissolved in acetone and applied as fumigants on *P. xylostella* adults.

Dose	KT_50_ ^4^/95% FL ^5^	Regression Equation	χ^2^	*p*-Value
LC_30_ ^1^	9.50 (9.16–9.87)	y = 7.586x − 7.418	3.664	0.300
LC_50_ ^2^	6.07 (9.16–9.87)	y = 11.307x − 8.852	6.193	0.103
LC_80_ ^3^	5.09 (4.65–5.50)	y = 10.365x − 7.323	7.847	0.049

^1^ LC_30_: 7 mg/mL. ^2^ LC_50_: 10 mg/mL. ^3^ LC_80_: 15 mg/mL. ^4^ KT_50_: Time to 50% knock-down. ^5^ FL: fiducial limits.

## Data Availability

Data are contained within the article.
